# Implementation of Home-Based Telerehabilitation of Patients With Stroke in the United States: Protocol for a Realist Review

**DOI:** 10.2196/47009

**Published:** 2023-07-11

**Authors:** Mohamed Mosaad Hasan, Miriam R Rafferty, Sara Tawfik, Ahmed Tawfik, Molly Beestrum, Justin D Smith, Lisa R Hirschhorn, Elliot J Roth, Donna M Woods

**Affiliations:** 1 Center for Healthcare Studies, Institute of Public Health and Medicine Feniberg School of Medicine Northwestern University Chicago, IL United States; 2 Shirley Ryan AbilityLab Chicago, IL United States; 3 Department of Physical Medicine and Rehabilitation Feinberg School of Medicine Northwestern University Chicago, IL United States; 4 Department of Medical Social Sciences Feinberg School of Medicine Northwestern University Chicago, IL United States; 5 Stritch School of Medicine Loyola University Chicago Maywood, IL United States; 6 The Kentucky College of Osteopathic Medicine University of Pikeville Pikeville, KY United States; 7 Galter Health Sciences Library & Learning Center Northwestern University Chicago, IL United States; 8 Department of Population Health Sciences Spencer Fox Eccles School of Medicine University of Utah Salt Lake City, UT United States; 9 Department of Psychiatry and Behavioral Sciences Feinberg School of Medicine Northwestern University Chicago, IL United States; 10 Department of Pediatrics Feinberg School of Medicine Chicago, IL United States

**Keywords:** telerehabilitation, telemedicine, stroke, implementation, realist review

## Abstract

**Background:**

Stroke is a common cause of mortality and morbidity. Insufficient and untimely rehabilitation has been associated with inadequate recovery. Telerehabilitation provides an opportunity for timely and accessible services for individuals with stroke, especially in remote areas. Telerehabilitation is defined as a health care team's use of a communication mode (eg, videoconferencing) to remotely provide rehabilitation services. Telerehabilitation is as effective as facility-based rehabilitation; however, it is infrequently used due to implementation barriers.

**Objective:**

The aim of the study is to explore the interaction between the implementation strategies, context, and outcomes of telerehabilitation of patients with stroke.

**Methods:**

This review will follow four steps: (1) defining the review scope, (2) literature search and quality appraisal, (3) data extraction and evidence synthesis, and (4) narrative development. PubMed via MEDLINE, the PEDro database, and CINAHL will be queried till June 2023 and supplemented with citation tracking and a gray literature search. The relevance and rigor of papers will be appraised using the TAPUPAS (Transparency, Accuracy, Purposivity, Utility, Propriety, Accessibility, and Specificity) and Weight of Evidence frameworks. The reviewers will extract and synthesize data iteratively and develop explanatory links between contexts, mechanisms, and outcomes. The results will be reported according to the Realist Synthesis publication standards set by Wong and colleagues in 2013.

**Results:**

The literature search and screening will be completed in July 2023. Data extraction and analysis will be completed in August 2023, and findings will be synthesized and reported in October 2023.

**Conclusions:**

This will be the first realist synthesis, uncovering the causal mechanisms to explain how, why, and to what extent implementation strategies impact telerehabilitation adoption and implementation.

**International Registered Report Identifier (IRRID):**

PRR1-10.2196/47009

## Introduction

### Telerehabilitation of Patients With Stroke

Stroke is a common cause of mortality and morbidity in the United States, affecting nearly 800,000 persons yearly [1]. Even though early rehabilitation of patients with stroke is pivotal for adequate recovery [2], rehabilitation is frequently delayed and underused [3]. Insufficient and untimely rehabilitation has been partly attributed to distance and transport challenges to rehabilitation facilities, especially for rural dwellers, inducing geographic disparity [4]. Nevertheless, both urban and rural residents experience various limitations to accessing facility-based rehabilitation: increasing rehabilitation costs, medical staff shortages, inadequate transportation systems and, after March 2020, pandemic-related contact restrictions [5,6]. In addition, more restriction to care is inflicted by the attitudinal, architectural, economic, and sensory barriers representing the omnipresent societal discrimination against people with disability [7].

Telerehabilitation is at least as effective as facility-based rehabilitation [8-10] and is typically less costly [11]. For that, home-based telerehabilitation is a promising solution that provides an opportunity for timely, effective, and accessible services to a large base of patients [12], especially in low-resource rural areas [13].

Multiple forms of technology provide a medium for telerehabilitation: telephone, messaging, email, videoconferencing, web-based platforms, robots, and virtual reality [14]. Currently, telerehabilitation via phone and videoconferencing is considered a basic technology [15,16]. Advanced technology includes virtual reality as it creates a physical experience with multiple sensory inputs: tactile, visual, auditory, and olfactory [12,17,18]. Robotics use in telerehabilitation is another advanced and evolving technology with great potential to enhance motor functions and measure patient progress [19].

### Telerehabilitation Implementation and the Interplay Between Context and Mechanisms

Most studies addressing telerehabilitation are feasibility and efficacy studies, leaving a gap in understanding the effective implementation of telerehabilitation [13,20]. Implementation science is “the scientific study of methods to promote the systematic uptake of research findings and other evidence-based practices into routine practice, hence aiming to improve the quality and effectiveness of health services” [21]. Many barriers hinder implementation: lack of knowledge and misbeliefs about the effectiveness of the intervention, inadequate planning for implementation, insufficient engagement of different stakeholders, and lack of resources, among others [22]. To overcome those barriers, implementation science pivots to designing and studying implementation strategies [23]. Implementation strategies are “methods or techniques to enhance a clinical program or practice’s adoption, implementation, and sustainability” [24]. Training, audit and feedback, coaching, and piloting are examples of implementation strategies.

While using the same strategies to enable implementation across various contexts is appealing, it brings limited success [25]. This is attributed to the multicomponent nature of implementation strategies and the complexity of the process of implementation that represents the interactions between the implementation strategy, the context of the setting in which implementation occurs, and the change process required for implementation [26,27].

Understanding the mechanisms of implementation strategies is vital to determine how and which strategies work and in what context for telerehabilitation [24,28,29]. Mechanisms are dynamic processes that drive and generate outcomes, often involving the understanding and reasoning of actors involved in implementation [30]. Establishing a telerehabilitation Community of Practice is an implementation strategy that might enhance telerehabilitation adoption; however, it does not directly result in adoption [31]. Through an underlying mechanism (eg, cognitive participation of the Community of Practice members), stakeholders may decide to adopt the change. Mechanisms are not observable by themselves, but observable data suggest their existence [31]. For example, increased adoption could be measured, but cognitive participation might be inferred to but not directly observed.

Context, Mechanisms, and Outcomes (CMO) configurations are at the heart of realist synthesis. CMO configurations are hypothetical until they are tested and found explanatory to the relationship between the strategies and the outcomes of interest [32]. CMO configurations explain what worked, how it worked, and what the involved mechanisms are [33]. Ultimately, researchers can respond to “will it work in a different context?” [33]. Realist researchers understand that implementation strategies work selectively, and outcomes vary in different settings [34,35]. Thus, recognizing implementation strategies patterns, as well as outcomes patterns, provide insight into the success or failure continuum [34].

This realist review aims to develop generalizable theories by examining each study to uncover the mechanism through which an implementation strategy works and the effects of various contexts on making it work [35]. The reviewers will untangle the complexity of implementation strategies through a frequent enhancement of the theoretical explanations of the empirical findings [36,37]. The output of this realist synthesis is a refinement of how the strategy “works, for whom, in what circumstances, in what respects and why?” [38].

The purpose of this realist review is to synthesize a theoretical explanation of the adoption and implementation of telerehabilitation of patients with stroke. For practitioners, this review will offer empirically rooted strategies for facilitating the implementation of telerehabilitation.

## Methods

### Study Design

This study will follow the guidelines proposed by Rycroft-Malone et al [39] and Pawson [34]. To explain how the implementation of telerehabilitation of patients with stroke operates, we will develop a middle-range theory (MRT), subject to refinement and validation as it can be used in the future implementation of telerehabilitation.

**Figure 1:**
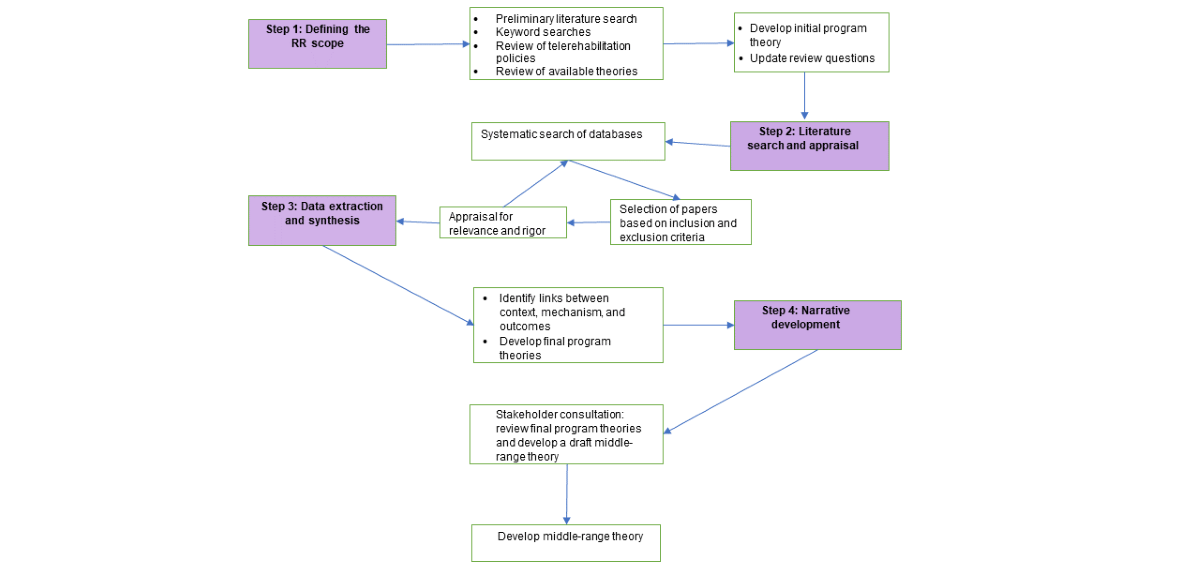
Flowchart of the RR steps. RR: realist review.

The review steps are (1) clarifying the scope and initial program theory (IPT) development, (2) searching for evidence appraising primary studies, (3) extracting data and synthesizing evidence, and (4) narrative development of conclusions. Figure 1 summarizes the main steps involved in this realist review.

### Step 1: Defining the Scope of the Review

The review team will formulate a descriptive and explanatory theoretical framework from the reviewed evidence. The focus of this review is on the implementation strategies of telerehabilitation. Implementation strategies are based on an initial practical understanding of the most effective way to deliver and implement an intervention, focusing on better implementation outcomes and addressing context-specific barriers to implementation.

Constructing a presynthesis conceptual framework marks the initial step in conducting a realist review [38]. This initial framework is referred to as IPT [40], which will provide an initial theoretical understanding of the implementation of telerehabilitation, necessary to explore implementation mechanisms. We will develop the IPT using concepts from multiple theoretical frameworks that researchers use for studying telemedicine adoption and implementation [41], namely: the Technology Acceptance Model, the Diffusion of Innovation Theory, the Theory of Interpersonal Behavior, the Perceived Usefulness, the Perceived Ease of Use, the Unified Theory of Acceptance and Use of Technology, and the Theory of Reasoned Action [42]. In addition, the review team will contribute their implementation science, telerehabilitation, and quality improvement knowledge and experience to refine the IPT [41]. The IPT will be refined and expanded as evidence arises throughout the iterative synthesis process [43]. The continuous iteration of the theory is essential to follow logic trails discovered in the literature to develop the understanding of telerehabilitation and its implementation.

### Step 2: Literature Search and Quality Appraisal

The literature search is iterative and directed to answer the questions that arise as the review progress [34,44]. To gain familiarity with the size and type of the available literature, one reviewer (MMH) will use *PubVenn* [45], which provides a graphical display of the extent of the literature, and *PubReMiner* [46], which analyzes the literature indexed in the PubMed MEDLINE database for frequent terms, journals, and authors.

The review team will use the 4 theoretical domains of the Realist Synthesis of Implementation Strategies (RES-IS) model to guide literature search and data extraction [39]. RES-IS is developed to research implementation strategies using the realist synthesis approach. It represents implementation strategies in 4 theoretical domains: properties of change agents, system change, technologies, and education interventions [39]. The change agent refers to the individuals’ attributes and the strategies they use to implement telerehabilitation successfully. Many terms are synonyms for change agents like facilitators, opinion leaders, knowledge brokers, champions, and experts. Systems changes refer to the organizational characteristics that facilitate the implementation and sustainment of telerehabilitation. Other terms related to systems change include social networks, culture, leadership, learning organizations, teamwork, and collaboration. Properties of technologies refer to the telerehabilitation technology itself and other technologies involved in facilitating its implementation. Education and training include academic detailing, reflection, action learning, and adult learning [39].

The context of implementation is at the center of the RES-IS model for its ubiquitous influence on the use and effect of strategies. In addition, an implementation strategy can target different levels: audits can induce changes in policies at the organizational level or alter local practices at the unit or individual level [47]. The term dose refers to the needed frequency and intensity of an implementation strategy (eg, training) to activate mechanisms that bring about outcomes [24,39].

The review team will use PubMed via MEDLINE, the PEDro database, a physical therapy evidence database, and CINAHL to conduct the topical search. A health information specialist (MB) leads the literature search. After reviewing the outcome of this topical search, we will rerun the search strategy, applying a search filter to limit the results to systematic reviews, logic models, and opinion pieces rich in concept-based discussions [48].

After identifying index papers, the review team will use the CLUSTER approach to search for Citations, Lead Authors, Unpublished Material, Scholar Searches, Theories, Early Examples, and Related Projects [48]. The CLUSTER search will be iterative and repeated to enhance the finding of relevant theories. The reviewers will also explore telerehabilitation-specialized journals (eg, *International Journal of Telerehabilitation*, *Smart Homecare Technology and Health*, and *Annual Review of Cybertherapy and Telemedicine*). As part of the CLUSTER approach, the reviewers will use the identified index documents to conduct a citation search of the index documents on Google Scholar using *Publish or Perish* software (Anne-Wil Harzing). To reach more resources, the reviewers will consult the American Telemedicine Association, the American Physical Therapy Association, and The American Occupational Therapy Association.

Documenting the literature search—The literature search is iterative and sometimes opportunistic [48]. The reviewers will use the EndNote software (Clarivate) [49] to manage the literature search process. To keep track of the literature search output, a new folder on EndNote will be added. Each literature search (a folder on EndNote) will document the sampling strategy, type of studies included, approaches, range of years, limits, inclusions and exclusions, terms used, and electronic sources [48,50].

The review team will use Rayyan (Oatar Computing Research Institute), a review management platform [51], to facilitate papers’ review and data extraction. To exclude irrelevant papers, 2 reviewers (MMH and AT) will screen the titles and abstracts. Based on the inclusion and exclusion criteria (Textbox 1), 3 reviewers (MMH, AT, and ST) will independently review the included full papers. The reviewers will meet regularly to discuss their decision on papers’ inclusion. Disagreement will be resolved by examining the paper’s language, focus, and inclusion criteria with another reviewer (MRR). Copies of papers or book chapters will be attached to the citation on Rayyan. Once saturation of the themes is achieved, the analysis of the included literature will conclude. Based on this analysis, the team may identify new inclusion criteria.

**Review inclusion and exclusion criteria.**

**Inclusion criteria**

- I—Intervention
  Home-based physical telerehabilitation of patients with stroke.
- S—Implementation strategy
  Implementation strategies for telerehabilitation of patients with stroke.
- C—Context
  Health care providers, for example, physicians, nurses, physiotherapists, and allied health professionals, are involved in home-based stroke telerehabilitation.
  Patients with stroke and their caregivers (eg, family members, professional caregivers, volunteers, and so on).
  Policy makers, decision makers, managers, leaders, and facilitators are involved in telerehabilitation.
  Any setting (eg, inpatient, outpatient, and home) involved in the implementation and delivery of telerehabilitation for patients with stroke.
  Studies based in the United States.
- M—Mechanisms
  Fundamental processes are involved in or responsible for action or reaction triggered by the implementation strategy.
- O—Outcomes
  Implementation outcomes guided by RE-AIM framework: Reach, Effectiveness of Implementation, Adoption, Implementation (fidelity and cost), and Maintenance. In addition, we will review Proctor’s implementation outcome framework: acceptability, appropriateness, feasibility, efficiency, speed, timeliness, and representativeness or equity related to reaching and adoption.

**Study design**

- All study designs will be included.
- Include empirical and nonempirical sources (eg, books, guidelines, policies, editorials, opinion papers, dissertations, among others).
- Other materials such as podcasts and recorded lectures as supplementary materials to published scientific evidence will be sought for insights.

**Exclusion criteria**

- Non-English papers.
- Studies that are done outside the United States.
- Studies that show low rigor.
- Papers address telerehabilitation in patients with conditions other than stroke (eg, postoperative rehabilitation) or other than in patients’ homes (eg, in the community).
- Papers with mere focus on cognitive and psychiatric rehabilitation of patients with stroke.
- Papers focused on the efficacy or effectiveness of telerehabilitation with no implementation-related details or insights.

The quality appraisal of the included papers will pass through 2 stages: the first stage will focus on relevance of the included papers to the objective of the review and the second one will evaluate their rigor. The review team developed criteria of relevance for this study (Textbox 2). The criteria for the relevance are to guide the team to include studies that can inform the MRT development [34].

**Criteria of study relevance to inform theory development.**

**High relevance**

- Describe contextual factors of implementation of telerehabilitation for patients with stroke.
- Describe stakeholders’ perspectives on the implementation of telerehabilitation.
- Describe implementation strategies of telerehabilitation for patients with stroke.
- Describe implementation outcomes of telerehabilitation for patients with stroke.
- Describe theories of implementation of telerehabilitation for patients with stroke

**Moderate relevance**

- Same as high relevance criteria but includes telerehabilitation for mixed patient populations that include other conditions in addition to stroke.

**Low to no relevance**

- Comparative studies of the effectiveness of telerehabilitation for patients with stroke that do not include implementation-related info.
- Describe the process of engineering the design of telerehabilitation technology for patients with stroke.
- Studies of prototypes and proofs-of-concept.

Assessment of rigor will be conducted through a combination of 2 different frameworks: TAPUPAS (Transparency, Accuracy, Purposivity, Utility, Propriety, Accessibility, and Specificity) framework, devised by Pawson et al [52], and Weight of Evidence (WoE) framework, devised by Gough [53,54].

TAPUPAS stands for Transparency, Accuracy, Purposivity, Utility, Propriety, Accessibility, and Specificity [52]. *Transparency* addresses whether the process of knowledge generation is open to external review and scrutiny [52]. Transparency extends beyond reporting research methods to how the researchers develop the research question, aims, and objectives [55]. *Accuracy* examines whether the researchers made accurate claims based on the results of their study [52,55,56]. *Purposivity* assesses the fit of the used methods for the purpose of the study [55]. Purposivity ensures the process of inquiry is methodologically sound and contextually appropriate. *Utility* considers the needs of the stakeholders and the fit of the study to satisfy those needs [56]. *Propriety* focuses on the ethical aspects of conducting the research [55]. *Accessibility* necessitates the dissemination and implementation of the research findings [55]. Both academic and nonacademic routes are appropriate for dissemination depending on who the end users are [55]. *Specificity* evaluates the methodological quality of the study and its conformance with the available standards [56].

WoE framework allows the opportunity to provide a grade for each of its 3 focus areas to be added up to an average grade for each paper [53]. WoE focus area A addresses the trustworthiness and coherence of the study, focus area B addresses appropriateness of the methods to the research question, focus area C assesses the relevance of evidence to the research question, and focus area D is the total grade based on the focus areas A, B, and C [53].

The focus area A of the WoE comprises 4 factors of the TAPUPAS framework: transparency, accuracy, accessibility, and specificity. The focus area B comprises purposivity. The focus area C comprises utility and propriety. Each focus area will be assigned a grade range from 1 (low quality), 2 (medium quality), or 3 (high quality) based on the TAPUPAS criteria within each of the WoE focus areas. For example, if a study is considered transparent and accurate, but it is not accessible or specific, it will receive a grade of 1.5 for WoE A [54]. The same logic will follow in the other focus areas. The score of WoE D will then reflect an overall average score, adding the scores of WoE A + B + C and dividing the total score by 3. Papers with WoE D score of 2 or more will be included. However, some papers with lower score, which provides important insight into the theory development will be included. In this case, the evidence generated out of inclusion of lower-quality studies will be marked as low-quality evidence.

### Step 3: Data Extraction and Evidence Synthesis

#### Overview

As this is an explanatory review that uses original papers to answer the study questions, a realist approach will be used for data extraction and synthesis. Data extraction will be conducted based on Multimedia Appendix 1 [1,2,5-42,57-59] forms. Essential text from the primary studies will be included to answer the review question [60]. All parts of the included papers will be examined to identify pertinent information. The reviewers (MMH and AT) will extract and code text from each of the papers. Then the coded texts will be synthesized together to search for a relationship between the intervention, implementation strategy, context, mechanisms, and outcomes (ISCMO) [61]. The identified intervention, ISCMO will consider different strategies and telerehabilitation modes. Discussions of the review team will guide the evidence synthesis, and each ISCMO configuration will be examined against the available empirical evidence [61].

#### Coding

A coding manual developed by May et al [62] will be used to guide the coding process. This manual is based on Normalization Process Theory (NPT), linking its 12 primary constructs to the CMO configurations [62]. NPT is a sociological theory that was developed to support studying and evaluating the implementation of complex health care interventions [62-64]. In addition, the constructs of the NPT have been mapped to the CMO configuration [62], rendering it a perfect fit for this realist review. Four NPT constructs conceptualize the context: strategic intentions, adaptive execution, negotiating capability, and reframing organizational logics. Another 4 constructs inform the mechanism of implementation: coherence building, cognitive participation, collective action, and reflexive monitoring. Lastly, outcomes include intervention mobilization, normative restructuring, relational restructuring, and sustainment. May et al [62] provided descriptions and examples of the NPT constructs as part of the coding manual (Multimedia Appendix 2).

Three reviewers (MMH, AT, and ST) will independently code the primary studies using NVivo (version 12; QSR International), a computer-assisted qualitative data analysis platform [65]. NVivo supports code-based inquiry, searching, and theorizing combined with the ability to annotate and edit documents [66]. Given the complex and iterative nature of conducting a literature search and synthesis in realist review, it is essential to prioritize and maintain transparency in methods to ensure the credibility and rigor of the review process [57], an endeavor NVivo will support [65]. In addition to detailing analytical microprocesses that lead to theory generation, another key feature of NVivo is its ability to integrate different data sets [65]. NVivo will be used early in the synthesis process. Each concept of the initial theory will be given a node in NVivo, and each node will be a hub for related materials used in the analysis. Each node will be linked to a memo to describe the review team discussions and thoughts behind concepts. For subconcepts, we will create child nodes. Concepts that are not supported by empirical evidence will be kept within the NVivo file as “unsubstantiated.” Once new evidence arises to support unsubstantiated concepts, we will bring those concepts back to the framework.

Coding will support the data extraction by assigning a salient and summative attribute to the extracted texts [58]. Data extraction and coding will link the primary studies, the research question, and the study findings [58]. Reviewers will meet regularly to discuss and consolidate the results.

Data will be combined and synthesized based on the following steps:

1. Three reviewers (MMH, AT, and ST) will organize extracted data into evidence tables; for example, to address the impact of systems characteristics on the implementation of telerehabilitation, the reviewers will form a table that includes the extracted data and source papers.
2. Three reviewers (MMH, AT, and ST) will independently identify themes from each paper. Themes are patterns across the included studies that are important to explain the implementation of telerehabilitation. Codes will then be refined based on discussions among the review team [59]. The same reviewers will meet regularly to discuss and challenge the themes.
3. Four reviewers (MMH, ST, AT, and MRR) will discuss and resolve differences, combine themes, and develop chains of inference (connections across papers linking the identified themes) through an iterative process [39]. The chains of inference will function as a basis for the ISCMO configurations,
4. Three reviewers (MMH, AT, and ST) will identify papers that contain the themes used to identify chains of inference.
5. Four reviewers (MMH, ST, AT, and MRR) will make connections among chains of inference, which will be an iterative process.
6. Four reviewers (MMH, ST, AT, and MRR) will use the chains of inference to generate ISCMO configurations confirmed by connecting back to the themes that emerged from the literature. The outcome of this step will be a comprehensive picture of implementation strategies, mechanisms, contexts, and outcomes chains [39,59]. The produced hypothesis will be used to create new program theories or refine them [39,59]. Then, we will provide a narrative based on the available evidence to support such a hypothesis.

Multimedia Appendix 3 contains outlines of the tables that will be used in each step.

### Step 4: Narrative Development

A narrative synthesis will summarize the nature of the implementation strategy, context, mechanism, and outcome configurations, and the supportive evidence from the primary studies. One reviewer (MMH) will take the lead in coordinating the presentation of the narrative synthesis using text, summary tables, and a logic model. After developing the theoretical framework, one reviewer (MMH) will write the review for publication. The origins of the inferences, the hypothesis, the developed MRT, and the source of the primary evidence on which they are based will be documented and defended. The reasoning process used to create the chains of inference, the hypotheses, and the final MRT will be documented and reported to enhance transparency [59].

### Ethics and Dissemination

Ethics approval is not required for this study as it is secondary research, synthesizing evidence from primary studies. The review team will disseminate the findings through peer-reviewed publications and conference presentations.

## Results

**Figure 2:**
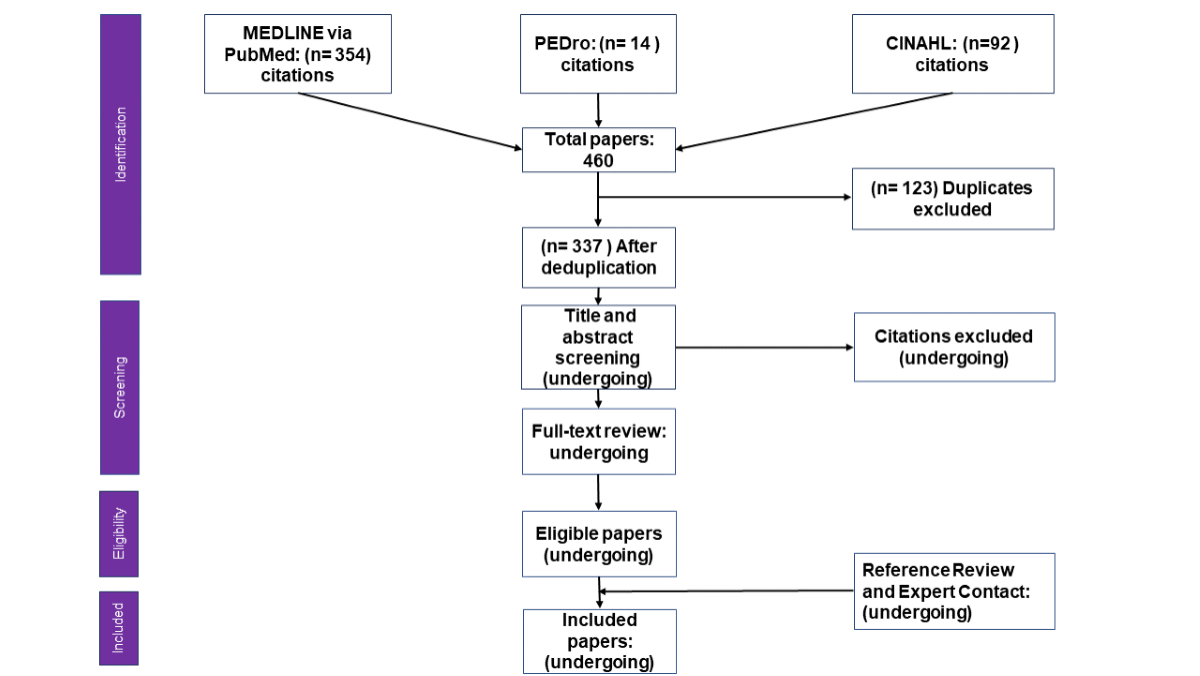
PRISMA (Preferred Reporting Items for Systematic Reviews and Meta-Analyses) flowchart.

Literature search and screening will be completed by the end of July 2023 and will be recorded in a PRISMA (Preferred Reporting Items for Systematic Reviews and Meta-Analyses) flowchart (Figure 2) [67]. A detailed search strategy is in Multimedia Appendix 4. Data collection will be based on the RES-IS model through forms that focus on the 4 domains (ie, properties of change agents, system change, technologies, and education interventions), their specific characteristics, and the impact of their interactions with the setting. Multimedia Appendix 1 gives more details on the planned data collection. The results of the review will be reported, following the RAMESES (Realist and Meta-Narrative Evidence Syntheses) standards (Multimedia Appendix 5) [36].

## Discussion

Telerehabilitation has the potential to bridge the access and timeliness gap of rehabilitation of patients with stroke. Yet, research on telerehabilitation implementation is still in its infancy. This review will enhance this field by developing a conceptual understanding of the interactions between different contexts and mechanisms of implementation. That will guide both implementation efforts at the practice level and future research at the academic level.

To our knowledge, this is the first realist review of the implementation of telerehabilitation for patients with stroke. A key strength of this review is that it will vet the causal mechanisms to explain how, why, and to what extent implementation strategies impact telerehabilitation implementation, a research gap not adequately explored. A further strength of the review is that it will examine the complex contexts in which various causal mechanisms are and are not activated. In addition, this review will inform future reviews by sharing our experience on using the recent NPT’s coding manual [62].

Nonetheless, this review has some limitations. First, the evidence will be dependent on the availability and quality of relevant useful published data. This means that this review’s findings and conclusions are limited to what have been published and it may restrict the ability to completely understand and describe the active causal mechanisms. To address this risk, the review team will use a comprehensive and iterative literature search that is expert-guided and source-diverse.

The potential impact of this review extends beyond academia and health care providers; policy makers stand to benefit from the insights gained as well. Delving into the causal mechanisms underpinning effective telerehabilitation implementation will yield essential information for designing experimental research studies that investigate how various strategies impact adoption, implementation, and sustainability. Armed with a deeper comprehension of these inner workings, decision makers can navigate adaptation efforts required for localizing policies pertaining to telerehabilitation implementation. This could lead to improved access and delivery of telerehabilitation services, ultimately resulting in better health outcomes for patients served by policy makers’ initiatives.

## Appendix

Multimedia Appendix 1 Data extraction.

Multimedia Appendix 2 Coding manual.

Multimedia Appendix 3 Evidence synthesis steps.

Multimedia Appendix 4 Search strategies.

Multimedia Appendix 5 Reporting guidelines.

## Abbreviations

CLUSTER: Citations, Lead Authors, Unpublished Material, Scholar Searches, Theories, Early Examples, and Related Project
CMO: Context, Mechanisms, and Outcomes
IPT: initial program theory
ISCMO: implementation strategy, context, mechanisms, and outcome
MRT: middle-range theory
NPT: Normalization Process Theory
PRISMA: Preferred Reporting Items for Systematic Reviews and Meta-Analyses
RAMESES: Realist and Meta-Narrative Evidence Syntheses
RES-IS: Realist Synthesis of Implementation Strategies
TAPUPAS: Transparency, Accuracy, Purposivity, Utility, Propriety, Accessibility, and Specificity
WoE: Weight of Evidence
