# Supplementary material for: Implementation of Home-Based Telerehabilitation of Patients With Stroke in the United States: Protocol for a Realist Review
Source: JMIR Res Protoc. 2023 Jul 11;12:e47009. doi: 10.2196/47009 (PMC10369311; doi:10.2196/47009)
Supplement: Multimedia Appendix 1 [file resprot_v12i1e47009_app1.docx]

**Data Extraction Form**

| **Full reference**: |
| --- |
| **Theory area 1 – Properties of change agent in telerehabilitation** |
| What are the characteristics of the change agent? |
|  |
| What impact do the characteristics of the change agent have on telerehabilitation? |
|  |
| What is the change agent? |
|  |
| What is the overall impact of the change agent intervention on telerehabilitation? |
|  |
| What is the interaction between the change agent and the setting? |
|  |
| What impact does the interaction between the change agent and the setting have on telerehabilitation? |
|  |
| **Is the evidence provided in this theory area good and relevant enough to be included in the synthesis (consider issues of sample size, data collection, data analysis, and claims made)** |
|  |
| **Theory area 2 –system change in telerehabilitation** |
| What are the characteristics of the systems change intervention(s)? |
|  |
| What impact do characteristics of the systems change intervention(s) have on telerehabilitation? |
|  |
| What is the system change intervention(s) used? |
|  |
| What is the overall impact of the system change intervention(s) used? |
|  |
| What is the interaction between the system change and the setting? |
|  |
| What impact does the interaction between the system change and the setting have on telerehabilitation? |
|  |
| What impact do senior leadership roles have in creating practice environments that integrate daily use of telerehabilitation at the point of care delivery? |
|  |
| **Is the evidence provided in this theory area good and RELEVANT ENOUGH to be included in the synthesis (consider issues of sample size, data collection, data analysis, and claims made)** |
|  |
| **Theory area 3 – properties of technologies used in telerehabilitation (technology = mechanisms not mediated through an INDIVIDUAL PERSON)** |
| What are the characteristics of the technological intervention(s)? |
|  |
| What impact do the characteristics of the technological intervention(s) have on telerehabilitation? |
|  |
| What is the technological intervention(s) used? |
|  |
| What is the overall impact of the technological intervention(s) used? |
|  |
| What is the interaction between the technological intervention and the setting? |
|  |
| What impact does the interaction between the technological intervention and the setting have on telerehabilitation use? |
|  |
| **Is the evidence provided in this theory area good and relevant enough to be included in the synthesis (consider issues of sample size, data collection, data analysis, and claims made)** |
|  |
| **Theory area 4 – education interventions in evidence-informed healthcare** |
| What impact do the characteristics of the education intervention(s) have in enabling telerehabilitation? |
|  |
| What are the characteristics of the education intervention(s)? |
|  |
| What is the education intervention(s) used? |
|  |
| What is the overall impact of the education intervention(s) used? |
|  |
| What is the interaction between the education intervention and the setting? |
|  |
| What impact does the interaction between the education intervention and the setting have on telerehabilitation use? |
|  |
| **Is the evidence provided in this theory area good and relevant enough to be included in the synthesis (consider issues of sample size, data collection, data analysis and claims made)** |
|  |
| **Is there evidence of particular theoretical perspective(s) impacting on the effectiveness of telerehabilitation?** |
|  |
| **Is there evidence of contextual factors impacting the effectiveness of telerehabilitation?** |
|  |
| **Is there evidence of the level of the intervention impacting the effectiveness of telerehabilitation?** |
|  |
| **Is there evidence of the intervention dose impacting the effectiveness of the intervention?** |
|  |
