# Supplementary material for: Implementation of Home-Based Telerehabilitation of Patients With Stroke in the United States: Protocol for a Realist Review
Source: JMIR Res Protoc. 2023 Jul 11;12:e47009. doi: 10.2196/47009 (PMC10369311; doi:10.2196/47009)
Supplement: Multimedia Appendix 3 [file resprot_v12i1e47009_app3.docx]

Step 1: Organizing extracted data from both reviewers into evidence tables

| **THEORY AREA 1 - What impact do the characteristics of the change agent have on telerehabilitation?** | |
| --- | --- |
|  |  |
|  |  |

Step 2: Themes from each article are identified by two reviewers (using the same table of step 1)

Step 3: Themes identified by different reviewers and amalgamated, and chains of inference are established

| **Author** | **Combined themes** | **Chain of inferences (connections across papers) to ultimately develop hypotheses** | **Decision** |
| --- | --- | --- | --- |
|  |  |  |  |

Step 4: Articles containing the themes used to derive the chains of inference are identified

| **Chains of Influence** | **Derived from the following themes in step 3** | **Articles** |
| --- | --- | --- |
|  |  |  |

Step 5: Making connections among chains of inference

Step 6: Generation of hypotheses using the chains of inference

| Hypotheses | Chain of Inference (theory level) | Chain of inference (sub-theory level) | Themes from the literature | Papers addressing the theme |
| --- | --- | --- | --- | --- |
