# Supplementary material for: Implementation of Home-Based Telerehabilitation of Patients With Stroke in the United States: Protocol for a Realist Review
Source: JMIR Res Protoc. 2023 Jul 11;12:e47009. doi: 10.2196/47009 (PMC10369311; doi:10.2196/47009)
Supplement: Multimedia Appendix 4 [file resprot_v12i1e47009_app4.docx]

Search Strategies for Medline

*Database: PubMed MEDLINE(R)*

Search Strategy:

(("Implementation Science"[Mesh] OR Implement* OR facilitat* OR barrier* OR decision-factor* OR decision-support* OR change-agent* OR knowledge-broker*)) AND (("Wireless technology"[MeSH] OR "Smartphone"[MeSH] OR "Telephone"[MeSH] OR "Telemedicine"[MeSH] OR "Telerehabilitation"[Mesh] OR Telemedicine* OR Tele-medicine* OR Tele-health OR Telehealth OR Smart-phone* OR Smartphone* OR Remote-consult* OR Video-call* OR Video-consult* OR Wireless-technolog* OR Video-communication* OR Wireless-system* OR Videoconferenc* OR Video-conferenc* OR E-health OR M-health OR Ehealth OR Mhealth OR Video-visit* OR Video-call* OR Video-based OR Tele-consult* OR Telehealthcare* OR Tele-monitor* OR Remote-monitor* OR patient-monitoring-device* OR HealthCall OR "interactive voice response") AND ("Stroke"[Mesh] OR "Brain Ischemia"[Mesh] OR stroke* OR brain-infarct* OR brain-ischem*) AND ("Home Care Services"[Mesh] OR home-based OR home-care OR remote*))

*Database: CINAHL*

Search strategy

((MH "Implementation Science") OR Implement* OR facilitat* OR barrier* OR decision-factor* OR decision-support* OR change-agent* OR knowledge-broker*)

AND

((MH "Wireless Communications") OR (MH "Videoconferencing") OR (MH "Telehealth") OR (MH "Teleconferencing") OR (MH "Cellular Phone") OR (MH "Smartphone") OR Telemedicine* OR Tele-medicine* OR Tele-health OR Telehealth OR Smart-phone* OR Smartphone* OR Remote-consult* OR Video-call* OR Video-consult* OR Wireless-technolog* OR Video-communication* OR Wireless-system* OR Videoconferenc* OR Video-conferenc* OR E-health OR M-health OR Ehealth OR Mhealth OR Video-visit* OR Video-call* OR Video-based OR Tele-consult* OR Telehealthcare* OR Tele-monitor* OR Remote-monitor* OR patient-monitoring-device* OR HealthCall OR "interactive voice response")

AND

((MH "Stroke+") OR (MH "Ischemic Stroke+") OR stroke* OR brain-infarct* OR brain-ischem*)

AND

((MH "Home Health Care") OR (MH "Home Rehabilitation") OR  home-based OR home-care OR remote*)

*Database: Pedro*

Search strategy: stroke home rehabilit* implement*
