# Supplementary material for: Implementation of Home-Based Telerehabilitation of Patients With Stroke in the United States: Protocol for a Realist Review
Source: JMIR Res Protoc. 2023 Jul 11;12:e47009. doi: 10.2196/47009 (PMC10369311; doi:10.2196/47009)
Supplement: Multimedia Appendix 5 [file resprot_v12i1e47009_app5.docx]

List of items to be included when reporting a realist synthesis

| **Item** | **Description** |
| --- | --- |
| *Title* |  |
| 1. Title | In the title, identify the document as a realist synthesis or review |
| *Abstract* |  |
| 2. Abstract | When acknowledging the publication requirements and house style, abstracts should ideally contain brief details of the study’s background, review question or objectives; the search strategy; the methods of selection, appraisal, analysis, and synthesis of sources; the main results; and implications for practice |
| *Introduction* |  |
| 3. Rationale for the review | Explain why the review is needed and what it is likely to contribute to the existing understanding of the topic area |
| 4. Objectives and focus of the review | State the objective(s) of the review either or the review question(s). Define and provide a rationale for the focus of the review |
| *Methods* |  |
| 5. Changes to the review process | Any changes made to the review process that was initially planned should be briefly described and justified |
| 6. Rationale for using realist synthesis | Explain why realist synthesis was considered the most appropriate method to use |
| 7. Scoping the literature | Describe and justify the initial process of exploratory scoping of the literature |
| 8. Searching processes | When considering the journal's specific requirements or another publication outlet, state and explain how the iterative searching was carried out. Provide details on all of the sources accessed for information in the review. When electronic searching databases have been carried out, the details provided should include the names of the databases, search terms, dates of coverage, and date last searched. If individuals familiar with the relevant literature either or topic area were contacted, indicate how they were identified and selected |
| 9. Selection and appraisal of documents | Explain how judgments were made about including and excluding data from documents and justify any decisions made |
| 10. Data extraction | Describe which data or information was extracted from the included documents and justify this selection |
| 11. Analysis and synthesis processes | Describe the analysis and synthesis processes in detail. This section should include information on the constructs analyzed and a description of the analytical processes |
| *Results* |  |
| 12. Document flow diagram | Provide details about the number of documents assessed for eligibility and included in the review, with reasons for exclusion at each stage and an indication of their source of origin (e.g., from searching databases, from reference lists). |
| 13. Document characteristics | Provide information on the characteristics of the documents included in the review |
| 14. Main findings | Present the key findings with a specific focus on theory building and testing |
| Discussion |  |
| 15. Summary of Findings | Summarise the main findings, taking into account the review’s objective(s),  research question(s), focus, and intended audience(s) |
| 16. Strengths, limitations, and future research directions | Discuss both the strengths of the review and its limitations. This should include (but need not be restricted to) (a) consideration of all of the steps in the review process and (b) comment on the overall strength of the evidence supporting the explanatory insights that emerged. The limitations identified may point to areas where further work is needed |
| 17. Comparison with existing literature | When applicable, compare and contrast the review’s findings with the existing literature (e.g., other reviews) on the same topic |
| 18. Conclusion and recommendations | List the main implications of the findings and place these in the context of other relevant literature. If appropriate, offer recommendations for policy and practice |
| 19. Funding | Provide details of funding source (if any) for the review, and the role played by the funder (if any), and any conflicts of interests of the reviewers |
